# The Puzzle of Italian Rice Origin and Evolution: Determining Genetic Divergence and Affinity of Rice Germplasm from Italy and Asia

**DOI:** 10.1371/journal.pone.0080351

**Published:** 2013-11-12

**Authors:** Xingxing Cai, Jing Fan, Zhuxi Jiang, Barbara Basso, Francesco Sala, Alberto Spada, Fabrizio Grassi, Bao-Rong Lu

**Affiliations:** 1 Ministry of Education, Key Laboratory for Biodiversity Science and Ecological Engineering, Department of Ecology and Evolutionary Biology, Fudan University, Shanghai, China; 2 Institute of Biophysics, National Research Council (CNR), Milano, Italy; 3 Department of Bioscience, University of Milan, Milano, Italy; Institute of Genetics and Developmental Biology, Chinese Academy of Sciences, China

## Abstract

The characterization of genetic divergence and relationships of a set of germplasm is essential for its efficient applications in crop breeding and understanding of the origin/evolution of crop varieties from a given geographical region. As the largest rice producing country in Europe, Italy holds rice germplasm with abundant genetic diversity. Although Italian rice varieties and the traditional ones in particular have played important roles in rice production and breeding, knowledge concerning the origin and evolution of Italian traditional varieties is still limited. To solve the puzzle of Italian rice origin, we characterized genetic divergence and relationships of 348 rice varieties from Italy and Asia based on the polymorphisms of microsatellite fingerprints. We also included common wild rice *O. rufipogon* as a reference in the characterization. Results indicated relatively rich genetic diversity (*H*_*e*_ = 0.63-0.65) in Italian rice varieties. Further analyses revealed a close genetic relationship of the Italian traditional varieties with those from northern China, which provides strong genetic evidence for tracing the possible origin of early established rice varieties in Italy. These findings have significant implications for the rice breeding programs, in which appropriate germplasm can be selected from a given region and utilized for transferring unique genetic traits based on its genetic diversity and evolutionary relationships.

## Introduction

Asian cultivated rice (*Oryza sativa* L., referred to as rice hereafter) is the third largest cereal crop of the world, and provides staple foods and nutrition for nearly one half of the global population [1]. Rice also has its important cultural and religious values (e.g., rice-wine and ceremonial applications) in many countries [2,3]. Sufficient and sustainable rice production is therefore crucial for the world food security. Germplasm in rice gene pool, particularly that represented by traditional varieties, plays an extremely important role in rice genetic improvement. The introduction of the “green-revolution” semi-dwarf trait identified from a traditional Chinese variety “Dijiaowujian” (Dee-Geo-Woo-Gen) to high yielding rice varieties has substantially increased the world rice production [4-6]. Understanding the history, origin, and evolutionary process of a particular set of rice varieties represented in a given geographical region will provide better opportunities for their efficient utilization as valuable germplasm in rice breeding.

 Rice was domesticated ~8000 years ago in the middle to lower parts of the Yangtze River region of China [7]. During the long-term domestication and cultivation, rice has adapted to various ecological conditions and evolved into two major ecotypes: *indica* and *japonica* [8,9]. The two ecotypes have significant genetic differentiation with considerable reproductive isolation [10]. Consequently, some scientists treated the two ecotypes as subspecies [8,10]. *Indica* rice is mainly found in tropical and subtropical rice planting regions, such as South Asia, Southeast Asia, and southern China; whereas *japonica* rice is mostly grown in temperate regions, such as East Asia, northern China, and southern Europe [8,11].

Italy is the largest rice production country in Europe, with about 250,000 hectares of cultivation area (www.enterisi.it) and 1.6 million tons of total grain production. Nearly all rice varieties grown in Italy are *japonica* ecotype [12], being adapted to the temperate climate. The efficient improvement of Italian rice varieties relies largely on the knowledge concerning the origin and evolutionary relationships of source germplasm used in breeding programs [13]. Such knowledge will provide breeders with information for the proper selection of alien varieties as parents in their hybridization programs. Determining the history and sources of rice varieties from Asia with diverse genetic relationships to Italian rice will facilitate the strategic utilization of innovative varieties (e.g., resistant to biotic and abiotic stresses) that have not yet been represented in Italy for rice improvement. In addition, understanding alien varieties that are highly similar or otherwise to local varieties with divergent genetic background may also help speed up the breeding process to reach the status of inbred lines through proper crosses and backcrosses. 

The first official documentation of rice introduction to Italy is related to the Spanish Presence in the Kingdom of Naples because of the ties between the Aragon family (The Kings of Naples) and the Sforza family (Dukes of Milan) in the second half of 15^th^ century [14]. Rice may have been introduced to Italy repeatedly in different periods of time *via* different routes such as by the Arabians or by Venetian commerce (e.g., The Travels of Marco Polo), although no written document about these is available. The earliest rice cultivation documented in Italy can be traced back to 1468 in the wetlands of Tuscany, near Pisa [15]. Rice cultivation expanded to ca. 20,000 ha around the Milanese area till 1700s [16]. At that time, the only rice cultivated in Italy was “Nostrale”, a variety susceptible to rice blast (*Magnaporthe oryzae*). To guarantee the continued rice cultivation that was seriously threatened by this fungal disease, new varieties were introduced from China and Japan at the beginning of 19th century. These varieties were characterized by their high yielding and resistance to rice blast. As a consequence, five Italian rice varieties were cultivated in Italy in 1872: Ostiglia, Bertone, Novarese, Francone, and Giapponese. Because of the recrudescence of blast attacks that created a new crisis for Italian rice cultivation, more rice varieties were imported from Asian countries including China, India, and Japan in 1880, which led to a substantial increase in the number of Italian rice varieties in the subsequent years. A milestone of Italian rice cultivation in history was the presence of a set of varieties grouped under the general name of “Chinese Originario” with high yield and strong resistance to blast [14]. However, there was no written document for the origin of these varieties, although the literal meaning of the name “Chinese Originario” is “originated from China”. 

Rice production in Italy fluctuated significantly, depending essentially on the national and international varieties used. A number of successful varieties (such as Balilla, Allorio, Pierrot, and Maratelli) were bred in 1926 through the systematic selection from “Chinese Originario” or, in a few cases, improved from old American varieties. This illustrates the importance of rice improvement in Italy relying on the introduction of alien germplasm from other countries. A number of recent studies have systematically characterized the existing Italian rice germplasm for its genetic diversity [17], agronomic traits and blast resistance [12], and temporal trend of variation [18]. Yet, no information is available about the origin and evolution of the Italian rice germplasm as a whole that has been extensively utilized. Such knowledge should be extremely valuable for rice breeding programs in Italy, quite apart from its cultural interests in understanding the naturalization of cultivated species in Europe in general.

To solve the “puzzles” of Italian rice origin, we characterized a large set of rice germplasm from extensive sources, based on the polymorphisms of microsatellite (SSR) fingerprints at 24 loci across the rice genome. The set of germplasm is represented by 183 Italian rice varieties, including nearly all traditional and modern varieties currently available in Italy, and 165 rice varieties from Asia. In addition, ten common wild rice (*Oryza rufipogon* Griff.) accessions were also used as a reference. The primary objectives of this study were to (i) estimate the general genetic diversity of different historical groups of Italian rice germplasm in comparison with that from other sources; (ii) determine genetic divergence of Italian rice germplasm from that of Asian rice germplasm; and (iii) trace the possible origin of Italian rice varieties based on genetic data. The answer of these questions will facilitate the continued genetic improvement of Italian rice for the sustainable rice production in the region.

## Materials and Methods

### Sampling of rice germplasm

A total of 348 rice varieties (*Oryza sativa*) from Italy and Asian countries were included in this study (Table 1; Table S1). Information concerning the *indica* or *japonica* characteristics, the accession number from germplasm banks, geographical locations of all the rice varieties was included in Table S1. Rice varieties from Italy were categorized into three groups based on the history of their genetic improvement. The first group (encoded as Italy-1) represented the Italian historical or traditional landraces that were directly used in the country long before the onset of rice genetic breeding programs, and consequently, these varieties *per se* have never been improved by genetic methods such as hybridization. The second group (Italy-2) represented the modern Italian varieties that were improved under local genetic breeding programs. The third group (Italy-3) included the modern Italian varieties improved under genetic breeding with rice germplasm from North America. These rice varieties covered the entire range of Italian rice cultivation regions and represented nearly all rice germplasm currently available in Italy (http://www.enterisi.it/ris_schede.jsp).

**Table 1:** Number of rice varieties and accessions of common wild rice (*O. rufipogon*) used in this study.

*Oryza* species analyzed	No. of Varieties/accessions	Group code	Country of origin
*O. sativa* L.	348		
	11	Italy-1	Italy (traditional varieties)
	122	Italy-2	Italy (improved varieties with local germplasm)
	50	Italy-3	Italy (improved varieties with N. American germplasm)
	130	China	China (from different provinces, see Table S1)
	20	E Asia	Japan, S. Korea
	6	S Asia	India, Sri Lanka
	9	SE Asia	Cambodia, Indonesia, Laos, Malaysia, Philippines, Vietnam
*O. rufipogon* Griff.	10	Wild	China, Guangdong and Hunan

Detail information including accession number and *indica*/*japonica* characteristics of each rice variety is included in Table S1.

Rice varieties from Asia were divided into four major groups according to their geographical origins: (1) the Chinese group that included varieties from different Provinces of this country (encoded as China); (2) the East Asian group included varieties from Japan and South Korea (E Asia); (3) the South Asian group included varieties from India and Sri Lanka (S Asia); and (4) the Southeast Asian group comprised of varieties from different countries in southeastern Asia (SE Asia), including Cambodia, Indonesia, Laos (Table 1; Table S1). In addition, ten accessions of common wild rice (*O. rufipogon*) from Guangdong and Hunan Province of southern China (Wild) were also included as a reference group (Table 1; Table S1). 

### DNA extraction, amplification, electrophoresis and SSR fragment score

Seeds of sampled rice varieties and wild accessions were germinated in an incubator at the temperature of ~25°C. The total genomic DNA was extracted from about 1g fresh leaf tissues for each seedling, separately, at about the three-leaf stage 10 days after seed germination. The extraction followed a modified cetyltrimethyl ammonium bromide (CTAB) protocol described by [19]. 

Twenty-four SSR primer pairs distributed on the two arms of each of the 12 rice chromosomes were selected for analyses from The RiceGenes Database (http://www.gramene.org). All the forward primers were labeled by one of the following fluorophore: FAM (blue), ROX (red), or JOE (green) (all fluorophore were from Invitrogen Inc., Shanghai, China). 

The polymerase chain reaction (PCR) was performed in the “2720 Thermal Cycler” (Applied Biosystems Inc., Foster, USA). Reactions were carried out in a volume of 10 μL mixture, containing 1×buffer (with Mg^2+^), 0.2mM each of dNTPs, 0.2μM each of SSR primers, 20 ng of genomic DNA and 0.3U of Taq polymerase (Takara Bio Inc., Otsu, Japan). The reaction procedure was programmed as following: a denaturation period of 4 min at 94°C followed by 28 cycles of 30 s at 94°C, 30 s at 55°C and 40 s at 72°C, and then 7 min at 72°C for the final extension.

According to the size differences of fragments amplified by each SSR primer pair, 3-5 amplified SSR products were added to the mixture of 9 μL Hi-Di Formamide (to denature the dsDNA) and internal lane size standard (GeneScan™ -500 LIZ) for fragment separation after denaturing at 94°C for 5 min and then cooled at 4°C. The SSR fragments were separated on a capillary electrophoresis genotyper, ABI 3130xl (Applied Biosystems Inc., Foster, USA). The separated SSR fragments were scored as genotypic data using the software Genemapper ver. 3.7 (Applied Biosystems Inc., Foster, USA).

### Data analysis

The genotypic data matrix from all rice varieties and wild accessions generated by the 24 SSR primer pairs was analyzed to estimate the level of genetic diversity. The following five diversity parameters were calculated: (1) the percentage of polymorphic loci (*P*); (2) the effective number of alleles per locus (*N*_*e*_); (3) the observed heterozygosity (*H*_*o*_); (4) Nei’s unbiased genetic diversity (*H*_*e*_) [20]; and (5) Shannon’s information index (*I*). In addition, the number of private alleles per rice group (*N*_*pa*_) was also calculated to estimate the uniqueness of each group. To determine genetic relationships among cultivated rice groups (see definition in sampling of rice germplasm) from various regions, the values of Nei’s unbiased genetic similarity [20] were calculated and compared. All above analyses were performed using the software GenAlEx ver. 6.2 [21].

To illustrate the relationships of different rice groups, an UPGMA dendrogram was constructed based on the unbiased genetic similarity [20], using the software NTSYS ver. 2.02 [22]. To estimate genetic relatedness of individual rice varieties from all regions, a neighbor-joining (NJ) tree was constructed based on a binary data set of 348 rice varieties, transformed from the genotypic data matrix. The combined data of wild rice accessions were used as an out-group in the NJ tree. The tree was constructed based on the “distance methods” installed in the software PAUP ver. 4.0 [23]. In addition, to determine the possible Italian rice origin, genetic similarity between the Italian traditional rice group (Italy-1) and each of the other rice groups from Asian regions, including China (that was further divided into subgroups representing various Provinces), E Asia, S Asia, and SE Asia was calculated. The level of genetic similarity between the Italy-1 group and other rice groups (and subgroups) was illustrated on a GIS map with a color-coded gradient with different intensity, applying the software ArcView GIS ver. 3.3.

## Results

### Genetic divergence of cultivated and wild rice from different regions

Rice germplasm included in this study with different origins presented a relatively high level of overall genetic diversity, although with a considerable degree of variation among rice groups (Table 2). A total of 442 alleles were identified in all cultivated rice across the 24 SSR loci. Percentage of polymorphic loci was 100% for all the 24 SSR loci in different cultivated rice groups (Table 2). The number of effective alleles per locus in cultivated rice groups varied from ~3.2 (Italy-1, Italy-2, and E Asia groups) to >5.2 (China group). Interestingly, much higher frequency of private alleles was found in some rice groups, such as China, Italy-2, and Italy-3 groups (Table 2). In general, a very low value (average 0.037) of observed heterozygosity was detected in the studied rice varieties, indicating the self-pollination feature of cultivated rice (Table 2). The overall genetic diversity of cultivated rice groups as estimated by the Nei’s unbiased expected heterozygosity [20] and Shannon’s information index (I) was ~0.75 and 1.90, respectively. However, the genetic diversity was not evenly distributed among the cultivated rice groups from different origins. The groups of cultivated rice represented by China, South Asia and Southeast Asia demonstrated a higher level of Nei’s unbiased expected heterozygosity (~0.76) than those represented by the three Italian groups (0.63-0.65). 

**Table 2:** Parameters of genetic diversity in cultivated rice from different regions based on 24 SSR loci.

Group code	***P***	***N_e_***	***H_o_***	***H_e_***	***I***	***N_pa_***
Italy-1	100.00%	3.163 (0.289)	0.053 (0.013)	0.654 (0.035)	1.215 (0.084)	0.042 (0.042)
Italy-2	100.00%	3.289 (0.332)	0.046 (0.007)	0.628 (0.036)	1.386 (0.094)	0.625 (0.189)
Italy-3	100.00%	3.221 (0.262)	0.041 (0.009)	0.634 (0.037)	1.382 (0.094)	0.667 (0.155)
China	100.00%	5.221 (0.517)	0.030 (0.006)	0.756 (0.028)	1.866 (0.103)	2.583 (0.500)
E Asia	100.00%	3.184 (0.233)	0.005(0.003)	0.677(0.018)	1.346 (0.052)	0.250 (0.132)
S Asia	100.00%	3.393 (0.209)	0.041 (0.017)	0.745 (0.026)	1.241 (0.062)	0.208 (0.085)
SE Asia	100.00%	4.019 (0.301)	0.039(0.021)	0.764(0.023)	1.443 (0.070)	0.375 (0.132)
Overall	100.00%	5.220 (0.547)	0.037 (0.005)	0.748 (0.029)	1.896 (0.102)	−

P: percentage of polymorphic loci; *N*_*e*_: the effective number of alleles; *H*_*o*_: the observed heterozygosity; *H*_*e*_: unbiased genetic diversity (Nei 1978); *I*: Shannon's information index; Numbers in parentheses indicate standard error; *N*_*pa*_: number of private alleles per variety group.

### Genetic relationships among different rice groups and varieties

The Nei’s unbiased genetic similarity [20] was calculated to estimate genetic relatedness of different groups of cultivated rice (Table 3). Results of Nei’s genetic similarity evidently indicated a close genetic relationship between Italy-1 and Italy-2 groups. These two Italian groups of rice varieties also shared a close genetic relationship with rice varieties from China (as a whole group) and Eastern Asian group. However, the Chinese rice group showed a relatively distant genetic relationship with the Italy-3 rice group that had North American germplasm. In addition, the three groups of Italian rice varieties showed relatively distant genetic relationships with those of South and Southeast Asian rice varieties.

**Table 3:** Pairwise unbiased genetic similarity (Nei, 1978) of the seven groups of rice varieties included in this study.

	Italy-1	Italy-2	Italy-3	China	E Asia	S Asia	SE Asia
Italy-1	—						
Italy-2	0.889	—					
Italy-3	0.688	0.795	—				
China	0.868	0.803	0.662	—			
E Asia	0.751	0.634	0.470	0.845	—		
S Asia	0.348	0.301	0.278	0.515	0.451	—	
SE Asia	0.498	0.472	0.387	0.604	0.561	0.685	—

**Figure 1:**
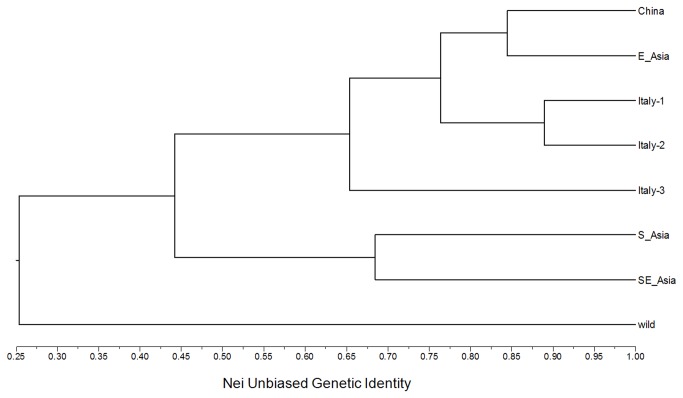
A UPGMA dendrogram indicates genetic relationships of cultivated rice groups with different origins (see Table 1 and Table S1), using wild rice as an out-group. The dendrogram was constructed based on the cluster analyses of Nei (1987) [20] unbiased genetic similarity of the rice groups.

For estimate genetic relationships of rice varieties from Italy (three groups) and Asia (four groups), a dendrogram (Figure 1) was constructed based on the UPGMA cluster analysis of Nei’s unbiased genetic similarity of all the cultivated rice groups and the wild rice accessions that was used as a reference group. The cluster analysis indicated an apparent separation of wild rice accessions from all cultivated rice groups, suggesting the significant genetic divergence of this wild ancestor from its cultigens. For the genetic relationships among different cultivated rice groups, the three groups of Italian cultivated rice varieties were clustered with those of China and East Asia, although the Italian traditional rice varieties (Italy-1) and locally improved varieties (Italy-2) were closely linked with Chinese and East Asian rice varieties (Figure 1), compared to the Italian improved rice varieties with North American germplasm (Italy-3) that had a relatively distant relationship with Chinese/East Asian and Italian traditional rice varieties. However, rice varieties from South Asia and Southeast Asia showed a considerably separated genetic status from all the other rice variety groups at the genetic similarity value of 0.44 (Figure 1).

For estimate genetic relationships of all the cultivated rice (348 varieties), a neighbor joining (NJ) tree was constructed based on the genetic distance of all the individual rice varieties using wild rice as an out-group (Figure S1). In general, results of the NJ tree did not indicate a clear separation of *indica* and *japonica* rice varieties as shown in previous studies. The NJ tree did not suggest an obvious variation pattern of the rice varieties from different regions either. However, the Italian traditional rice varieties categorized in Italy-1 (highlighted with a yellow color in Figure S1) showed their close relationships with some Chinese rice varieties (highlighted with a green color in Figure S1) in different clusters. For example, the Italian variety ENRCRR739 was closely associated with Chinese varieties AAV002902, AAV002901, and AAV002890 from Hebei Province, and Italian variety ENRCRR229 was also closely associated with Chinese varieties AAV002881 and AAV003125 from Hebei and Guangdong Provinces (for details see: Table S1, Figure S1).

### Genetic relationships of Italian traditional rice with Chinese rice from different Provinces

**Figure 2:**
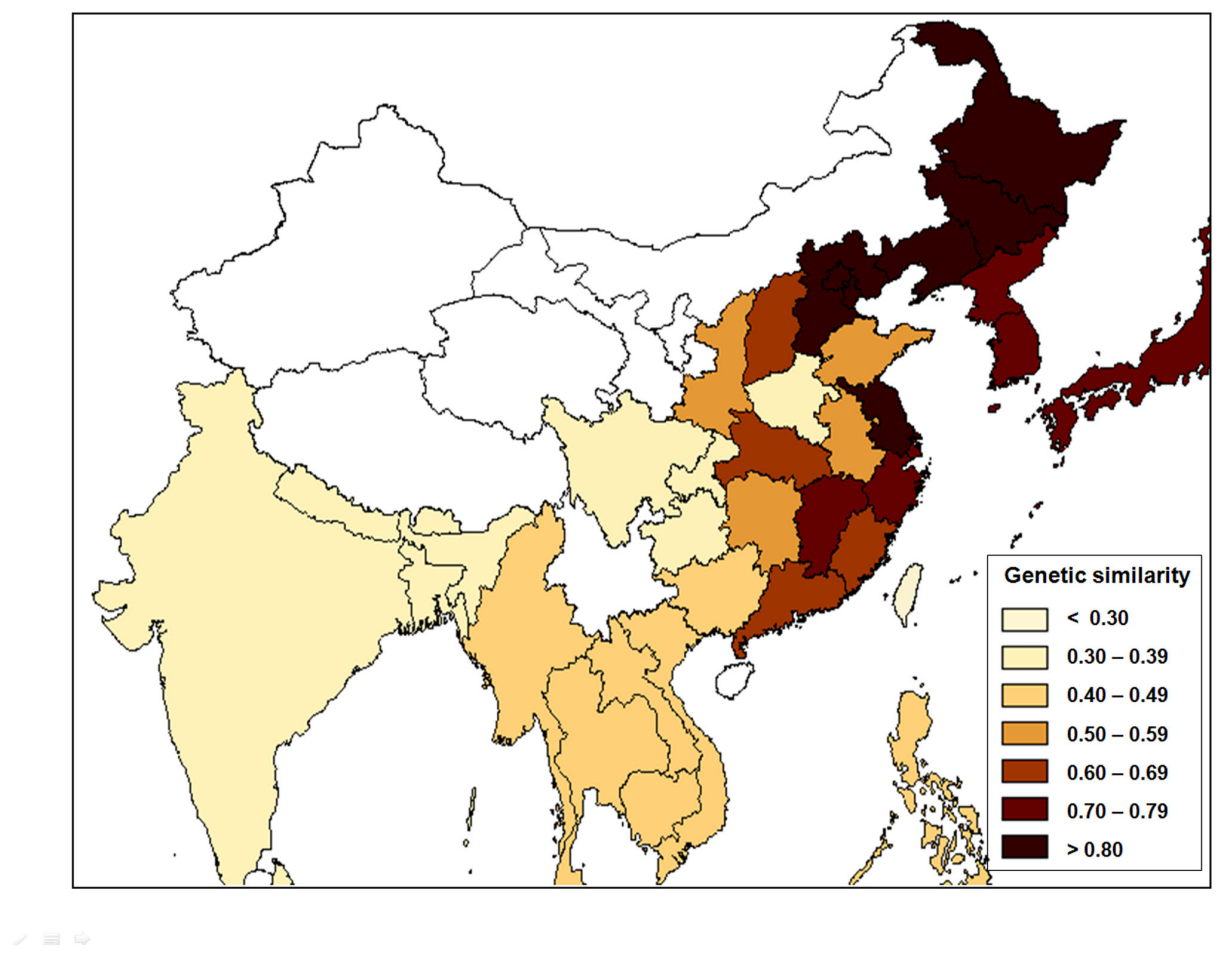
A GIS (geographical information system) map illustrating the genetic similarity of the Italian traditional rice verities (Italy-1) to those from Asia. Rice varieties from China were further divided into subgroup represented by Provinces (see Table S1). The intensity (seven levels) of color-codes indicates the degree of relatedness as estimated by the Nei (1987) [20] unbiased genetic similarity. The legend at the right-down corner indicates the level of genetic similarity.

The above results demonstrated a very close genetic relationship of Italian traditional rice varieties (Italy-1) with Chinese and East Asian varieties as groups. Given the abundant genetic diversity in Italian traditional rice varieties (with only a few varieties, see Tables 2 and 3) that well represented the level of diversity in the entire Italian rice germplasm, it was possible to use Italian traditional and Asian rice varieties for estimating their genetic relatedness and the origin of Italian rice. As the largest rice-growing country, China used different types (e.g., *indica* and *japonica*) of rice varieties in different regions, which was considered for dividing the whole group into subgroups. The newly divided Asian regions included different Provinces of China, Southeast Asia, East Asia, and South Asia (Table 1, Table S1). Consequently, genetic similarity between Italian traditional rice (Italy-1 group) and varieties represented by different groups and subgroups from Asia was calculated to determine the possible geographical origin of Italian cultivated rice. According to the values of genetic similarity, the relationship of Italian traditional rice varieties to those from other regions explained above was arbitrarily divided into seven levels (see legend in Figure 2). The results showed that rice varieties from these Chinese Provinces of Heilongjiang, Liaoning, Jilin, Hebei, and Jiangsu had the highest level of genetic similarity (>0.8) with Italian traditional rice (Figure 2), indicating the closest genetic relationship of the northern-based Chinese rice varieties with the Italian rice. Rice varieties from other Chinese Provinces, such as Zhejiang, Jiangxi, as well as those from East Asia showed the second highest level (0.7-0.79) of genetic similarity with Italian rice. The lowest genetic similarity was observed between Italian traditional rice varieties and those from South Asia, Southeast Asia, and some Chinese Provinces, such as Henan, Sichuan, and Guizhou (Figure 2). 

## Discussion

### Genetic variation of rice germplasm from Italy and Asia

We analyzed a large set of rice germplasm including 183 varieties representing nearly all Italian rice germplasm, 165 varieties from Asia, and ten accessions of wild rice (*O. rufipogon*) as a reference, to estimate its genetic variation and relatedness. The total number of alleles (442) detected in these varieties was unexpectedly high, compared with those detected in a previous study where 172 Italian rice varieties and 47 introduced foreign varieties were analysed using 24 SSR loci [12]. Interestingly, the significantly higher number of private alleles found in Chinese rice varieties provided important information on the uniqueness of rice germplasm from China. The value of observed heterozygosity (~0.04) for cultivated rice is in agreement with that reported by [12], which confirms the self-pollination mating system of cultivated rice, compared with the wild ancestor *O. rufipogon* that had a much higher *H*_*o*_ value (<0.2, data not shown). 

The level of genetic diversity (*H*_*e*_, 0.63-0.65) in Italian rice varieties (three groups) is comparable to that (0.59) reported in a previous study on the temporal trends of variation in Italian germplasm [18]. However, the diversity level of Italian rice varieties was considerably lower than that of the overall (~0.75) rice germplasm included in this study, and was also lower than that of rice varieties from China (~0.76), Southeast Asia (0.76), South Asia (0.75), and East Asia (0.68). These results strongly suggest the potential value of Asian rice germplasm for the genetic improvement of Italian rice varieties. The previous study that demonstrated the stability of genetic diversity in Italian rice germplasm over the last two centuries [18] is likely due to two important reasons considered the results from this study. First, the early introduced (or traditional) Italian rice varieties are composed of an essential set of germplasm with abundant genetic diversity, which is reflected by the fact that only a small number (11) of varieties represented the equivalent amount of genetic diversity in all available rice varieties in Italy. It is therefore important to safeguard the Italian traditional rice varieties for the production and improvement of modern rice varieties in Italy and the neighbouring regions, apart from their unique quality and values as a staple food. Second, exotic rice germplasm is constantly introduced to Italy in breeding programs, which may have largely compensated the possible losses, if any, of genetic variation in Italian rice. All these results together indicate that continued introduction of elite rice germplasm from Asia, particularly from China, SE Asia, and S Asia should be prioritized for further genetic improvement of rice varieties in Italy, as well as in the neighbouring European countries.

### Genetic relationship of Italian rice and its implications in breeding

Results from this study demonstrated a clear genetic relationship among the three sets of Italian rice germplasm, and that between Italian and Asian rice germplasm. The Italian traditional (Italy-1) and locally improved modern (Italy-2) rice varieties share a close affinity, and showed their close linkage with rice varieties from China and East Asia. This finding reveals that the Italian rice varieties produced based on germplasm from similar sources (such as Italy-1 and Italy-2) remain much closer genetic relationships than those with germplasm from different sources such as North American (Italy-3). In addition, Italian rice germplasm as a whole showed a relatively distant genetic affinity with that from South Asia and Southeast Asia, where *indica* varieties are essentially grown. Given the fact that Italy basically uses *japonica* rice varieties[12], it makes sense that Italian rice germplasm shares a close relationship with that from China (particularly north parts) and East Asia. This emphasizes the important role of environmental conditions in introducing exotic genetic resources for breeding or direct production in a target region. 

Notably, the Italian traditional and locally improved rice varieties have a close genetic affinity with Chinese and East Asian varieties. When alien germplasm was introduced from North America and used as breeding materials for hybridization, the genetic relationship between Italy-3 and Chinese/East Asian rice varieties became loose due to mixture of North America rice germplasm. Obviously, the evolution and divergence of Italian rice germplasm depend essentially on the introduced exotic rice varieties from different geographical sources and the breeding methods. The NJ tree based on genetic relatedness of individual rice varieties did not show particular groupings of these varieties associated with either *indica* and *japonica* differentiation as suggested by other studies [24,25], or geographical distribution. This clearly reflects the complex situation of multiple introduction of rice germplasm from different sources to Italy and various breeding methods adopted in this country [12,18]. However, the close relationship of the Italian traditional rice with some Chinese varieties provide useful information for tracing the origin and evolution of Italian rice, although the resolution of such a relationship based only on 24 SSR markers is quite limited. 

Understanding genetic relationships of the current Italian rice germplasm has its important implications in rice breeding, through properly selecting and introducing exotic germplasm into Italian rice varieties [17]. The introduction of exotic rice germplasm closely related to Italian rice varieties can facilitate the easiness of hybridization between the Italian local varieties and the exotic germplasm due to limited reproductive barriers, facilitating more successful hybridization. On the other hand, the introduction of distantly related and diverse alien germplasm may create novel genetic recombinants and/or specific genotypes in hybrid progeny and broaden genetic background of the Italian local rice germplasm.

### The possible origin of early established Italian rice

Our results generally indicated a close relationship of Italian traditional and locally bred modern varieties to Chinese varieties as a whole group. However, China is a large country with the extensive cultivation of diverse types of *indica* and *japonica* rice varieties [9,23,24]. It is therefore interesting to identify the particular region, from which the “ancestor” of the Italian currently used rice germplasm was possibly originated or first introduced. 

Our results based on genetic similarity analysis of Italian traditional varieties (Italy-1) with Chinese rice germplasm represented by different Provinces evidently indicated their close genetic affinity to that from northern rice cultivation regions (Figure 2). These regions include Hebei, Liaoning, Jilin, and Heilongjiang Provinces, where the climate conditions are relatively similar to those of Italian rice cultivation regions. Results from the NJ tree also provide some clues of such genetic affinities between individual varieties from Italy and China (Figure S1). These results suggest that the Italian traditional rice was most likely introduced to Italy from northern China for the first time. This finding can well explains the reason that the rice varieties imported to Italy in the 1880s from Asian region were collectively referred to as “Chinese originario” that replaced the early introduced Italian rice varieties such Nostrale. Undoubtedly, these rice varieties have contributed significantly to Italian rice production in the historical time.

Although the tales such as Marco Polo’s contribution to the introduction of rice from China to Italy may never be fully confirmed, results from this study provide solid genetic evidence to confirm the close linkage between Italian and Chinese rice varieties. Rice varieties from northern China may provide genetically most accessible and useful germplasm for Italian rice breeding because of their close genetic relatedness and abundant genetic diversity, even though rice varieties from other regions in Asia can also provide unique and especially valuable germplasm that has not been represented in the Italian rice gene pool. 

## Conclusions

As the largest rice production country in Europe, Italy holds important and diverse rice germplasm, which guarantees the sustainable rice production and breeding in Italy and neighboring countries. Knowledge concerning the genetic diversity, origin, and evolutionary relationships of Italian rice has important implications for its effective breeding by selecting proper exotic rice germplasm. The early introduced rice varieties such as “Chinese originario” have played an important role in Italian rice production and breeding programs. However, the origin and evolutionary relationships of these early introduced varieties have not been properly documented. Results based on SSR fingerprints of a large set of rice germplasm from Italy and Asia in this study demonstrate a close genetic relationship between Italian and Chinese rice varieties, suggesting the possible origin of Italian traditional rice from northern China. Although the conclusion on Chinese origin of Italian traditional rice still needs supports from further studies, this finding has significant implications for Italian rice improvement by including appropriate exotic germplasm in its breeding programs.

## Supporting Information

Figure S1 **A Neighbor-Joining (NJ) tree showing individual-based genetic relationships of 348 rice varieties analyzed in this study (see Table 1 and Table S1 for detail information), using wild rice as an out-group (root).** The yellow-color highlighted entries indicate the Italian traditional varieties, and the green-color highlighted entries indicate Chinese varieties. The NJ tree was constructed based on the “distance methods” installed in the software PAUP ver. 4.0 [23].
(PDF)

Table S1 **Rice germplasm included in the analysis.** Accession numbers and *indica* (i) or *japonica* (j) characteristics are indicated in the parentheses following the name of a rice variety. IBS = Institute of Biodiversity Sciences of Fudan University; AAV = Shanghai Agricultural Gene Center; ENR-CRR = Ente Nazionale Risi-Centro Ricerca sul Riso; RNV = Registro Nazionale Varietà 2013.
(DOC)
