# Supplementary figures and images for: The Puzzle of Italian Rice Origin and Evolution: Determining Genetic Divergence and Affinity of Rice Germplasm from Italy and Asia
Source: PLoS One. 2013 Nov 12;8(11):e80351. doi: 10.1371/journal.pone.0080351 (PMC3827184; doi:10.1371/journal.pone.0080351)

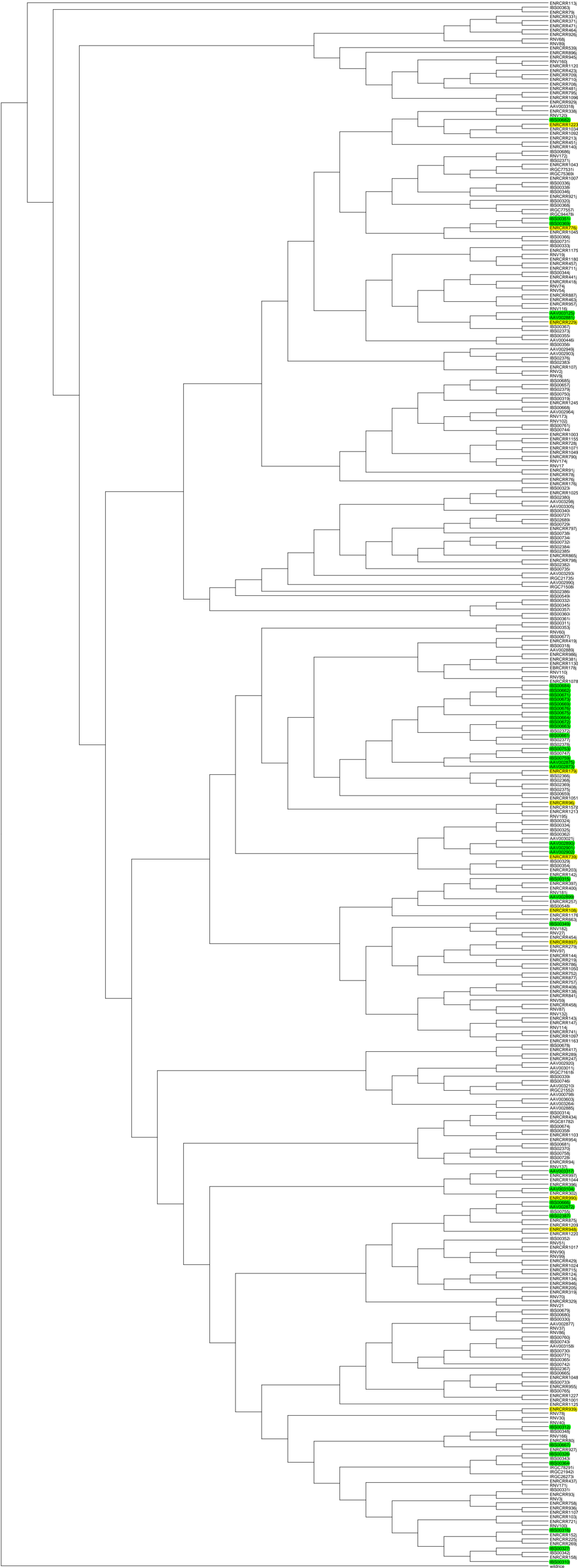

Supplement: Figure S1 — A Neighbor-Joining (NJ) tree showing individual-based genetic relationships of 348 rice varieties analyzed in this study (see Table 1 and Table S1 for detail information), using wild rice as an out-group (root). The yellow-color highlighted entries indicate the Italian traditional varieties, and the green-color highlighted entries indicate Chinese varieties. The NJ tree was constructed based on the “distance methods” installed in the software PAUP ver. 4.0 [23]. (PDF) [file pone.0080351.s001.pdf]
