# Supplementary material for: The Puzzle of Italian Rice Origin and Evolution: Determining Genetic Divergence and Affinity of Rice Germplasm from Italy and Asia
Source: PLoS One. 2013 Nov 12;8(11):e80351. doi: 10.1371/journal.pone.0080351 (PMC3827184; doi:10.1371/journal.pone.0080351)
Supplement: Table S1 — Rice germplasm included in the analysis. Accession numbers and indica (i) or japonica (j) characteristics are indicated in the parentheses following the name of a rice variety. IBS = Institute of Biodiversity Sciences of Fudan University; AAV = Shanghai Agricultural Gene Center; ENR-CRR = Ente Nazionale Risi-Centro Ricerca sul Riso; RNV = Registro Nazionale Varietà 2013. (DOC) [file pone.0080351.s002.doc]

**Table S1.** Rice germplasm included in the analysis. Accession numbers and *indica* (i) or *japonica* (j) characteristics are indicated in the parentheses following the name of a rice variety. IBS = Institute of Biodiversity Sciences of Fudan University; AAV = Shanghai Agricultural Gene Center; ENR-CRR = Ente Nazionale Risi-Centro Ricerca sul Riso; RNV = Registro Nazionale Varietà 2013

| Group code | No.of  Cultivars / accessions | Name of cultivars | Origin |
| --- | --- | --- | --- |
|  |  |  |  |
| Italy-1 | 11 | Allorio precoce (ENR-CRR 96,j), Americano-1600 (ENR-CRR 108,j), Balilla (ENR-CRR179,j), Bertone (ENR-CRR 229,j), Lencino (ENR-CRR 739,j), Maratelli (ENR-CRR 776,j), Ostiglia (ENR-CRR 897,j), Pierrot (ENR-CRR 939,j), Precoce6 (ENR-CRR 948,j), Ranghino (ENR-CRR 990,j), Vialone nero (ENR-CRR 1223,j) | Italy (traditional cultivars) |
|  |  |  |  |
| Italy-2 | 122 | Adelaide Chiappelli (ENR-CRR 76,j), Agostano (ENR-CRR 80,j), Alba (ENR-CRR 91,j), Alfa (ENR-CRR 93,j), Alice (ENR-CRR 94,j), Alpe (ENR-CRR 103,j), Ambra (ENR-CRR 107,j), Andolla (ENR-CRR 113,j), Arborio (ENR-CRR 124,j), Arborio Precoce (ENR-CRR 134,j), Arco (ENR-CRR 138,j), Ares (ENR-CRR 143,j), Argo (ENR-CRR 144,j), Ariete (ENR-CRR 147,j), Arpa (ENR-CRR 152,j), Asso (ENR-CRR 157,j), Augusto (ENR-CRR 158,j), Baldo (ENR-CRR 176,j), Bali (ENR-CRR 178,j), Balzaretti (ENR-CRR 203,j), Baraggia (ENR-CRR 205,j), Bastia (ENR-CRR 213,j), Belgioso (ENR-CRR 219,j), Bravo (ENR-CRR 257,j), Brio (RNV 30,j), Carmen (RNV 40,j), Carnaroli (ENR-CRR 279,j), Cervo (ENR-CRR 289,j), Chimera (ENR-CRR 302,j), Cobra (RNV 51,j), Corbetta (ENR-CRR 331,j), Costella (ENR-CRR 319,j), Cripto (ENR-CRR 338,j), Dedalo (ENR-CRR 371,j), Delfino (RNV 59,j), Diana (ENR-CRR 381,j), Dorella (ENR-CRR 396,j), Doria (ENR-CRR 397,j), Drago (ENR-CRR 400,j), Elba (ENR-CRR 417,j), Elio (ENR-CRR 418,j), Elvo (ENR-CRR 419,j), Ercole (RNV 70,j), Europa (ENR-CRR 429,j)), Eurosis (RNV 74,j), Flipper (ENR-CRR 441,j), G. Vercelli (ENR-CRR 451,j), Garda (ENR-CRR 458,j), Genio (RNV 87,j), Ghibli (ENR-CRR 463,j), Giada (ENR-CRR 464,j), Gigante (RNV 90,j), Ibis (RNV 95,j), Idra (ENR-CRR 539,j), Italico (ENR-CRR 663,j), Karnak (RNV 97,j), Koral (RNV 99,j), La Ferla (ENR-CRR 715,j), Lago (ENR-CRR 728,j), Lido9 (ENR-CRR 741,j), Lomellino (ENR-CRR 752,j), Lord (ENR-CRR 757,j), Loto (ENR-CRR 758,j), Marengo (ENR-CRR 786,j), Marte (RNV 110,j), Medusa (ENR-CRR 790,j), Miara (ENR-CRR 797,j), Mida (ENR-CRR 798,j), Minerva (RNV 114,j), Nembo (ENR-CRR 841,j), Novara (ENR-CRR 865,j), Nuovo Maratelli (RNV 120,j), Oldenico (ENR-CRR 875,j), Onda (ENR-CRR 877,j), Originario Lencino (ENR-CRR 887,j), Oscar (ENR-CRR 896,j), Padano (ENR-CRR 921,j), Perla (ENR-CRR 927,j), Petaso (ENR-CRR 926,j), Piemonte (ENR-CRR 936,j), Porto (ENR-CRR 945,j), Poseidone (ENR-CRR 946,j), Precoce Rossi (ENR-CRR 954,j), Prever (ENR-CRR 955,j), Primo (RNV 137,j), Prometeo (ENR-CRR 957,j), Raffaello (ENR-CRR 986,j), RB (ENR-CRR 997,j), Redi (ENR-CRR 1001,j), Reno (ENR-CRR 1003,j), Ribe (ENR-CRR 1007,j), Ringo (ENR-CRR 1017,j), Riva (ENR-CRR 1024,j), Rizzotto (ENR-CRR 1025,j), Roma (EMR-CRR 1034,j), Romeo (ENR-CRR 1043,j), Roncarolo (ENR-CRR 1045,j), Roncolo (ENR-CRR 1048,j), Rosa Marchetti (ENR-CRR 1049,j), Rova (ENR-CRR 1050,j), Roverbella (ENR-CRR 1051,j), San Pietro (ENR-CRR 1078,j), Sant Andrea (ENR-CRR 1071,j), Sara (ENR-CRR 1092,j), Savio (ENR-CRR 1097,j), Selenio (RNV 116,j), Senatore Novelli (ENR-CRR 1103,j), Sereno (ENR-CRR 1107,j), Smeraldo (ENR-CRR 1125,j), Spina (ENR-CRR 1130,j), Stirpe 136 (ENR-CRR 1155,j), Strella (ENR-CRR 1163,j), Stresa (RNV 172,j), Tea (RNV 174,j), Tosca (RNV 181,j), Ulisse (RNV 182,j), Vega (ENR-CRR 1209,j), Veneria (ENR-CRR 1213,j), Vialone Nano (ENR-CRR 1220,j), Volano (ENR-CRR 1227,j), Zena (ENR-CRR 1245,j), Zeus (RNV 195,j) | Italy (improved cultivars) |
|  |  |  |  |
| Italy-3 | 50 | Adelio (ENR-CRR 78,j), Adriano (ENR-CRR 79,j), Aiace (RNV 2,j), Albatros (RNV 3,j), Apollo (RNV 9,j), Ardizzone (ENR-CRR 140,j), Arelate9ENR-CRR 142,j), Artemide (RNV 17,j), Asia (RNV 19,j), Bengal_ENR-CRR 225,j), Bianca (RNV 27,j), Bonnet Bell (ENR-CRR 247,j), Cadet (ENR-CRR 269,j), Cesare (RNV 78,j), Condor (ENR-CRR 329,j), Creso (RNV 54,j), CRLB1 (RNV 37,j), Deneb (RNV 60,j), Ebro (ENR-CRR 408,j), Ellebi (RNV 68,j), Eolo (ENR-CRR 423,j), Fenis (ENR-CRR 437,j), Fragrance (ENR-CRR 434,j), Galileo (ENR-CRR 454,j), Gange (ENR-CRR 457,j), Gemini (RNV 86,j), Giano (RNV 89,j), Giove (ENR-CRR 471,j), Gladio (ENR-CRR 481,j), L201 (ENR-CRR 708,j), L202 (ENR-CRR 709,j), L203 (ENR-CRR 710,j), L204 (ENR-CRR 711,j), Lady Wright (ENR-CRR 721,j) , Lamone (RNV 100,j), Libero (RNV 102,j), Mercurio (ENR-CRR 795,j), Perseo (ENR-CRR 929,j), Pony (RNV 132,j), Romolo (ENR-CRR 1044,j) , Saturno (ENR-CRR 1096,j), Scudo (RNV 160,j), Sillaro (ENR-CRR 1120,j), SIS-R215 (RNV 166,j), Sprint (RNV 171,j), Tanaro (RNV 173,j), Tebonnet (ENR-CRR 1175,j), Tejo (ENR-CRR 1176,j), Thai (ENR-CRR 1180,j), Titano (RNV 21,j) | Italy (improved cultivars with North America germplasm) |
|  |  |  |  |
|  |  |  |  |
| China | 130 |  | China (from different provinces) |
| Anhui | (5) | Anxuan4 (IBS00734,i), Hehuadao (IBS00765,j), Laohonggu (IBS00334,j), Zhongjingdao (IBS00336,j), Zhongxian58 (IBS00733,i) |  |
| Fujian | (4) | Baikehong (IBS00343,i), Heimaodadongnuo (IBS00344,j), Huangzhinuo (IBS00346,j), Laobaishu (IBS00345,i) |  |
| Guangdong | (9) | Aijiaonante (AAV000446,i), Chuanzhanchi (AAV003125,j), Danuo (IBS00354,j), Guluai4 (IBS02689,i), Huangdaozhan (IBS00352,i), Menjiaoying (IBS00353,j), Ribengu (IBS00351,i), Xinaozhan2 (IBS00738,i), Zaoshuiyinzhan (IBS00355,i) |  |
| Guangxi | (3) | Guangtounuo15-3 (IBS00358,i), Liushirizao (IBS00356,i), Yinsibai (IBS00357,i) |  |
| Guizhou | (3) | Baikeshangu (AAV003293,i), Shiyangnuo (AAV003305,j), Yanzhan (AAV003298,j) |  |
| Hebei | (12) | Acxiaohongmang (AAV002872,j), Fndahongmang (AAV002885,j), Fnxiaobairen (AAV002875,j), GYDDdahongmang (AAV002902,j), Huaianshuidao (AAV002901,j), Laishuimaiweidao (AAV002903,j), Lldabaimang (AAV002881,j), Qianxibendidaozi (AAV002873,j), Tschaoxiandao (AAV002889,j), Wnhongmangdao (AAV002890,j), Ytxiaohongmang (AAV002877,j), Zhululaozu (AAV002899,j) |  |
| Henan | (2) | Deguodao (IBS00368,j), Zijindou (IBS00369,j) |  |
| Heilongjiang | (9) | Fujing3 (IBS00666,j), Ha3632 (IBS00663,j), Hailin1 (IBS00320,j), Kongyu131 (IBS00665,j), Kenjiandao9 (IBS00657,j), Mudanjiang19 (IBS00664,j), Puyou10 (IBS00662,j), Songjing7 (IBS00661,j), Xinxuan1 (IBS00659,j) |  |
| Hubei | (7) | Bailianzhan (IBS00744,i), Bawangbian2 (IBS00363,j), Dongtingwanxian (IBS00743,i), Hongxinuo (IBS00362,i), Lichuandabaigu (IBS00360,i), Longxuzhan (IBS00361,i), Nantianzhan (IBS00742,i) |  |
| Hunan | (8) | Bashizao (IBS00365,i), Gaoshanhong (IBS00364,i), Guidongxian (AAV003158,i), Xianghenuo (IBS00366,j), Xianghu115-29 (IBS00755,j), Xiangwanxian5 (IBS00735,i), Yuchi231-8 (IBS00729,i), Zaodanuo (IBS00367,j) |  |
| Jiangsu | (9) | Baimangjing (IBS00327,j), Jiangyinzao (AAV002949,j), Manyedao (AAV002964,j), Nanjing7 (IBS00758,j), Taihujing2 (IBS00753,j), Tutounuodao (AAV002990,j), Wujiangxian (IBS00326,i), Wuyujing3 (IBS00759,j), Yelipan (IBS00329,j) |  |
| Jiangxi | (6) | Danuo (IBS00342,j), Gannong3 (IBS00338,i), Ganzhou80zao (IBS00746,i), Jianyanzao (AAV003104,j), Jinbaoyin (IBS00340,i), Xuguzao (IBS00339,i) |  |
| Jilin | (12) | Dangdibeihaidao (IBS00319,j), HJTguangluyudao (IBS00318,j), Ji01E262 (IBS00672,j), Ji99F90 (IBS00671,j), Jiudao39 (IBS00674,j), Jiudao47 (IBS00673,j), Suijing4 (IBS00667,j), Tong95-74 (IBS00676,j), Tongjing611 (IBS00675,j), Tongyin58 (IBS00668,j), Tongyu124 (IBS00669,j), Zaoshengjingzu (IBS00316,j) |  |
| Liaoning | (12) | Dan9877 (IBS00684,j), Dongshi8 (IBS00677,j), Gaoyou35 (IBS00678,j), Jing9540 (IBS00680,j), Liaodong128 (IBS00679,j), Liaojing294-4 (IBS00682,j), Liaojing727 (IBS00681,j), Liaonong2096 (IBS00686,j), Weiguo (IBS00312,j), Wulong99 (IBS00685,j), Xinyouzaosheng (IBS00315,j), Yuanchao (IBS00314,j) |  |
| Shaanxi | (2) | Madaozi (AAV003317,j), SCGjiangmidao (AAV003318,j) |  |
| Shandong | (2) | Hongjiangmi (IBS00348,j), Huludaozi (IBS00349,j) |  |
| Shanxi | (2) | Bendidao (IBS00310,j), Maodao (IBS00311,j) |  |
| Sichuan | (2) | Xiangu (AAV003264,i), Xiaogu (AAV003210,i) |  |
| Shanghai | (8) | Baomanuo (AAV003603,j), Dongyangxian (IBS00323,i), Huangdao (IBS00760,j), Huangzhong (IBS00325,j), Jifeijie (IBS00771,j), Laohuzhong (IBS00761,j), Miziwan (AAV002920,j), Yazuizhongjing (IBS00324,j) |  |
| Taiwan | (3) | C712001 (IBS00730,i), Jianongxian6 (IBS00731,i), Jianongxianyu7 (IBS00732,i) |  |
| Zhejiang | (10) | Aiyangdao (IBS00330,j), Baimier (IBS00332,i), Guanglixian (IBS00331,i), Jia935 (IBS00727,i), Laohudao (IBS00333,j), Xianghu25 (IBS00747,j), Xueliqing, (AAV003011,j) Yongjing194 (IBS00750,j), Yuanfengzao (IBS00728,i), Zihong (AAV003021,j) |  |
|  |  |  |  |
|  |  |  |  |
| E Asia | 20 | Chaoxi290 (IBS02366,j), Chaoxi850 (IBS02367,j), Cheolweon32 (IBS02386,i), Daobei42 (IBS02368,j), Daobei44 (IBS02369,j), Deyu20 (IBS02375,j), Donghaechal (IBS02379,j), Fushi139 (IBS02370,j), Fuxi3937 (IBS02373,j), Iri385 (IBS02378,j), Nonglinnuo203 (IBS02372,j), Ryongsung6 (IBS02376,j), Suweon287 (IBS02382,i), Suweon309 (IBS02383,i), Suweon310 (IBS02384,i), Suweon360 (IBS02380,j), Suweon381 (IBS02385,i), Sak Zo Si (IBS02387,i), Unbong8 (IBS02377,j), Zidao (IBS02371,j) | East Asia:  Japan, S. Korea |
|  |  |  |  |
|  |  |  |  |
| S Asia | 6 | ADUKKAN (IRGC 81782,i), ARC11669 (IRGC 21552,i), ARC11909 (IRGC 21735,i), ARC12148 (IRGC 21942,i), BG951 (AAV000798,i), SMV11 (IRGC 77531,i) | South Asia:  India, Sir Lanka |
|  |  |  |  |
| SE Asia | 9 | A Tiya (IRGC 94478,i), Ba Danh (IRGC 78291,i), Batu (IRGC 71508 ,i), IR36 (IBS00549,i), IR64 (IBS00548,i), Mrc172-9 (IRGC 26273,i), Neangourk (IRGC 75369,i),  Pare Buri-Buri-2 (IRGC 77557,i), Sibungkuk (IRGC 71618,i) | Southeast Asia: Cambodia, Indonesia, Laos, Malaysia,  Philippines, Vietnam |
|  |  |  |  |
| Wild | 10 | *Oryza rufipogon* (IBS07000-IBS07009) | China: Guangdong and Hunan Provinces |
|  |  |  |  |
